# Association between the healthy eating index and sarcopenia in Chinese elderly: a cross-sectional study

**DOI:** 10.1186/s12877-025-06143-w

**Published:** 2025-07-05

**Authors:** Jianghua Huo, Chongyu Ding, Ya-Qian Xu, Hui Zhang, Yulu Gong, Darong Hao, Jing Wang, Hongyan Duan, Xiangwei Li

**Affiliations:** 1 School of Public Health and Management, Jiangsu Medical College, Yancheng, Jiangsu 224005 China; 2 School of Global Health, Chinese Centre for Tropical Diseases Research, Shanghai Jiao Tong University School of Medicine, Shanghai, 200025 China; 3 School of Public Health, Shanghai Jiao Tong University School of Medicine, Shanghai, 200025 China; 4 Department of Geriatric Medicine, Henan Provincial People’s Hospital, Zhengzhou, Henan China; 5 Hainan International Medical Center, Shanghai Jiao Tong University School of Medicine, Hainan, 571434 China

**Keywords:** Healthy eating index, Sarcopenia, Older population, CLHLS

## Abstract

**Background:**

Sarcopenia, characterized by the loss of muscle mass and strength, is a major health concern among older adults. It increases the risk of disability and mortality. While nutrition is a key factor in preventing sarcopenia, the relationship between diet quality and sarcopenia, especially through the Healthy Eating Index (HEI), remains limited and inconsistent.

**Methods:**

We conducted a cross-sectional analysis of data from the 2018 wave of the Chinese Longitudinal Healthy Longevity Survey (CLHLS) to examine the association between HEI and sarcopenia in adults aged 60 years and older. HEI scores were derived from the consumption frequency of 13 food groups collected through face-to-face interviews. Sarcopenia was assessed using the SARC-F questionnaire. Multivariable logistic regression models were fitted to estimate the association.

**Result:**

Among 14,257 Chinese elders, HEI was inversely associated with the prevalence of sarcopenia. A 1-unit increase in HEI corresponded to a 1.2% reduction in the prevalence of sarcopenia (OR = 0.988, 95% CI: 0.979–0.998, *P* = 0.014). Compared to the elderly in the first quartile of HEI, multivariable-adjusted ORs for those in the second to fourth quartiles were 0.934 (95% CI: 0.796–1.095), 0.839 (95% CI: 0.709–0.992), and 0.772 (95% CI: 0.641–0.930). Subgroup analysis revealed heterogeneous associations across different sub-populations. Restricted cubic spline analysis showed a negative and linear relationship between HEI and sarcopenia in the overall population (P-overall = 0.049, P-nonlinear = 0.971), which remained significant in males, females, and those aged 75 years or older.

**Conclusion:**

Better diet quality, reflected by higher HEI scores, is associated with a lower prevalence of sarcopenia in older adults, highlighting the importance of nutrition in preventing sarcopenia.

**Supplementary Information:**

The online version contains supplementary material available at 10.1186/s12877-025-06143-w.

## Introduction

Sarcopenia, defined as a progressive and pervasive age-related syndrome characterized by the decline in both muscle mass and function, poses a significant public health challenge in the aging population [1]. Studies have reported that sarcopenia is associated with increased morbidity and mortality from various adverse health outcomes, including falls, fractures, reduced quality of life, and depression [2, 3]. As the global proportion of elderly individuals continues to rise rapidly [4], the prevalence of sarcopenia in community-dwelling older adults has been reported to range from 10 to 32% [2, 5, 6], further underscoring its growing impact as a public health concern.

Nutrition is widely recognized as playing a critical role in the prevention of sarcopenia [7]. Several studies have explored the relationship between specific nutrients or food groups-such as protein, vitamin D, vegetables, and fruits-and sarcopenia [7–9]. The latest version of the Healthy Eating Index (HEI), developed by the U.S. Department of Agriculture, incorporates independent evaluations of 13 dietary components (e.g., added sugars and saturated fats) and serves as a comprehensive tool for assessing overall diet quality [10, 11]. Although HEI has been closely linked to various nutrition-related health outcomes, research on its association with sarcopenia remains limited and inconsistent [12–14]. For instance, one study found that the Alternative Healthy Eating Index-2010 (AHEI-2010) was inversely associated with sarcopenia risk in women, whereas no significant association was observed in men or the general population [14]. In contrast, another study suggested that adherence to healthy eating could enhance handgrip strength, and that HEI-2015 was negatively correlated with probable sarcopenia [13].

To fill this gap, we conducted this cross-sectional study, using data from 2018 wave of Chinese Longitudinal Healthy Longevity Survey (CLHLS), to explore the relationship between HEI and sarcopenia among the Chinese elderly population.

## Methods

### Study population

CLHLS is an extensive and ongoing longitudinal study on the determinants of healthy longevity in China. The CLHLS utilized a multistage stratified cluster sampling approach, implemented across 22 provinces selected from China’s 31 provincial administrative divisions. A total of 631 municipal and county units were randomly chosen through this framework, collectively encompassing approximately 85% of the national population [15].

All participants provided paper-based informed consent before data collection. During the data collection procedure, only the participant and the interviewer were present. The study was approved by the Biomedical Ethics Committee of Peking University, Beijing, China (IRB00001052–13074) [15].

**Fig. 1:**
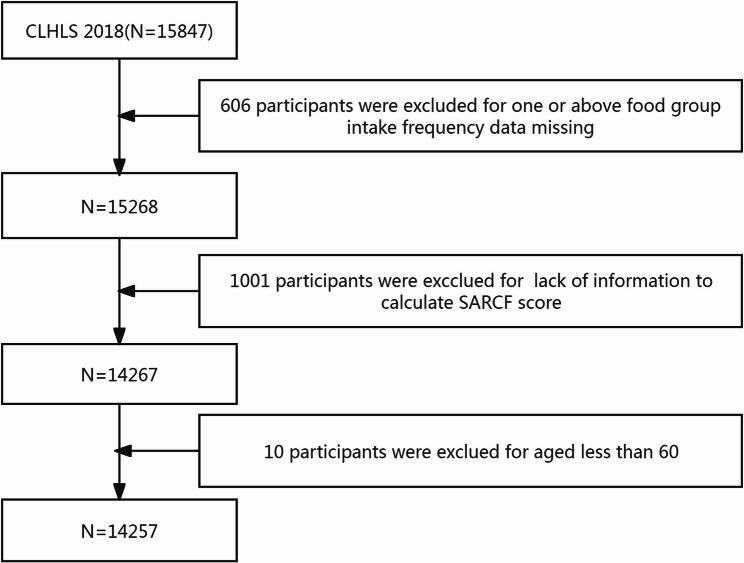
Flowchart of participants included in the CLHLS 2018

This cross-sectional study utilizes data from the 2018 wave of CLHLS, which was conducted between 2017 and 2018 and comprised of a total of 15,874 respondents, with 12,411 of them being newly interviewed in 2018. The dataset was freely downloaded from Peking University Open Research Data (http://opendata.pku.edu.cn/). After excluding participants without complete dietary information to calculate the healthy eating index and those with missing data on key variables for assessing sarcopenia, a total of 14,257 participants aged 60 years and older were included. The detailed flowchart is shown in Fig. 1.

### Calculation of the healthy eating index HEI

The HEI score was calculated based on previously established methods with modified according to the CLHLS dietary information [10, 16, 17]. The current consumption frequency of 13 food groups, used to construct the HEI, was collected through face-to-face interviews by trained interviewers using structured food frequency questionnaire. These food groups included vegetables, fruits, meats, fish, eggs, beans and their products, tea, garlic, nuts, mushrooms or algae, dairy products, salt-preserved vegetables, and sugar.

Scores were assigned to each food group’s consumption frequency as follows: For vegetables and fruits, scores ranged from 0 to 3, with “rarely or never” scoring 0, “occasionally” scoring 1, “except in winter” scoring 2, and “almost every day” scoring 3. For the other 11 food groups, scores were assigned based on the frequency of consumption as follows: “rarely or never” scored 0 points, “not every month, but occasionally” scored 1 point, “not every week, but at least once a month” scored 2 points, “not every day, but at least once a week” scored 3 points, and “almost every day” scored 4 points, while sugar and salt-preserved vegetables were reverse scored [16].

Finally, the score of each food group was summed together to obtain the HEI score of participants. The HEI score ranged 0 ~ 50 with the higher HEI score representing a healthier diet.

### Assessment of sarcopenia

As described in previous studies in CLHLS [18], the SARF-C questionnaire, a widely used screening tool for sarcopenia with good internal consistency reliability (Cronbach’s α varied from 0.76 to 0.81) in three cohorts and low sensitivity (13.7–37.9%) but high specificity (94.8–98.1%) in Chinese community elders [19–21], was employed in this study. The questionnaire consists of five questions assessing strength, assistance walking, rising from a chair, climbing stairs, and falls, as described previously with slight modifications [22].

In brief, in the CLHLS, the strength was measured by the question, “Are you able to carry a 5 kg weight?” Assistance walking was assessed by the question, “Are you able to walk one kilometer?” Climbing stairs was not directly measured in the CLHLS but was substituted with the question, “Are you able to crouch and stand three times?” to assess lower limb performance. For both the strength and walking questions, scores were assigned as follows: 2 points for “yes,” 1 point for “a little difficult,” and 0 points for “unable to do so.”

Rising from a chair was assessed by the question, “Are you able to stand up from sitting in a chair?” with 2 points for “yes, without using hands,” 1 point for “yes, using hands,” and 0 points for “no.” Falls were measured by the number of falls in the past year, with 2 points assigned for four or more falls, 1 point for 1 to 3 falls, and 0 points for no falls.

The total SARC-F score ranges from 0 to 10, with respondents considered to have sarcopenia with the SARC-F score ≥ 4 in this study [22, 23].

### Potential covariables

Potential confounding factors, which include socio-demographic characteristics (sex, age, residence, co-residence type, economic status, marital status, education level) and health-related factors (smoking, drinking, physical activity, health status, body mass index [BMI], and chronic disease status), were selected based on prior evidence of their associations with sarcopenia and diet, and were adjusted to improve the accuracy of the results [2, 7]. The adjusted social-demographic factors and some health-related factors, including smoking, alcohol consumption, physical activity and history of diseases were collected by trained interviewer through face-to-face interview.

Age were categorized into two groups:<75 years and ≥ 75 years. Co-residence type was categorized into two groups: “alone” for participants who lived alone, and “not alone” for those who lived in an institution or with household members. Education level was categorized into four groups based on the years of education: Illiterate (0 years), Primary school (1 ~ 6 years), Middle school (7 ~ 9 years) and High school or above (≥ 10 years). Marital status was stratified into two groups: “currently married and living with spouse/cohabiting” and “separated/divorced/widowed/never married.” Economic status was categorized as “difficulty,” “average,” or “wealthy” according to participants’ responses to the question, “How do you rate your economic status compared with other local people?” Smoking, drinking, and exercise habits were classified into three groups: “never,” “former,” and “current.” Health status was assessed and stratified into three distinct categories by trained interviewers: (1) “surprisingly healthy” for participants reporting no chronic conditions and maintaining full functional independence; (2) “relatively healthy” for those with only minor ailments but preserving basic daily functioning; and (3) “ill” for individuals with moderate to major degrees of major ailments or illnesses or with significant functional impairments. BMI was calculated as weight (kg)/[height (meter)]^2^ and divided into four groups(underweight [< 18.5 kg/m^2^], normal [18.5 kg/m^2^ ≤ BMI < 24 kg/m^2^], overweight [24.0 kg/m^2^ ≤ BMI < 28.0 kg/m^2^] and obesity[BMI ≥ 28 kg/m^2^]) according to China Working Group criteria [24]. A history of hypertension is asserted for participants with SBP ≥ 140 mmHg and (or) DBP ≥ 90 mmHg, or who have been diagnosed by a doctor or are currently taking medication for hypertension. A history of diabetes, heart disease, stroke, and cancer is asserted if participants have been diagnosed by a doctor or are currently taking medication for these conditions.

### Statistic methods

Demographic characteristics of the study subjects were summarized using standard descriptive methods. HEI scores were analyzed both as a continuous variable and as quartiles (Q1-Q4). Variance analysis or Kruskal-Wallis test was used for continuous variables and the chi-square test was used for categorical variables to compared the difference between quartiles of HEI.

Three logistic regression models were built to explore the association between HEI and sarcopenia. Specifically, Model 1 was the crude model, Model 2 adjusted for sociodemographic factors, including age, sex, residence, co-residence type, economic status, marital status, and education level. Model 3 additionally included smoking, drinking, physical activity, health status, BMI and history of diseases. Model diagnostics including Hosmer-Lemeshow goodness-of-fit test and Nagelkerke’s R² were performed to confirm model appropriateness [25].

In addition to analyzing HEI scores as a continuous variable, the association was also assessed using HEI quartiles (Q1-Q4) as a categorical variable. A trend test (P-trend) was performed with the medium of the HEI within each category to assess whether the association showed a significant decreasing trend across increasing HEI quartiles [26]. Odds ratios (ORs) and 95% confidence intervals (CIs) were calculated to quantify the associations. Finally, subgroup analysis was performed with multiple logistic regression models to evaluate the consistency of observed results between different predefined subgroups, considering potential effect modifier such as age, gender, marital status, Residential area, co-residence type, economic status and physical activity.

Missing values in covariates were handled using ‌Markov Chain Monte Carlo (MCMC)-based multiple imputation‌. Five imputed datasets were generated with 20 iterations, and pooled estimates (OR and 95% CI) were derived via Rubin’s rules to minimize bias [27].

Restricted cubic splines (RCS) were utilized to investigate potential non-linear relationships between HEI and prevalence of sarcopenia. In the spline models, the 10th percentile of the ln-transformed HEI distribution was set as the reference value (OR = 1.00), with knots at the 5th, 35th, 65thand 95th percentiles and adjusted for covariables in model 3 [28].All statistical analyses were conducted using SPSS version 26.0 (IBM Corp., Armonk, NY, USA) and R version 4.0.0 (R Foundation for Statistical Computing, Vienna, Austria). Statistical significance was defined as a two-tailed *P*-value < 0.05.

## Results

### Characteristics of participants

The characteristics of the 14,257 participants in this study are presented in Table 1, stratified by quartiles of the HEI score: Q1 (≤ 20), Q2 (21–25), Q3 (26–30), and Q4 (≥ 31). Overall, 43.89% were male, and 78.22% were aged 75 years or older. Approximately 40.35% were married or cohabiting, and 44.26% lived in rural areas. Nearly one-third (30.15%) engaged in regular physical activity, while about one in seven were current smokers (14.76%) or drinkers (14.04%).

**Table 1:** Characteristics of participants from CLHLS 2018 according to HEI score

	Total (*n* = 14257)	Q1 (*n* = 3279)	Q2 (*n* = 3819)	Q3 (*n* = 3501)	Q4 (*n* = 3658)	*P*-value
**HEI**, **median (IQR)**	26 (21–31)	17 (15–19)	23 (22–24)	28 (27–29)	34 (32–37)	
**Sarcopenia**						< 0.001
No	8412 (59.00)	1594 (48.61)	2170 (56.82)	2125 (60.70)	2523 (68.97)	
Yes	5845 (41.00)	1685 (51.39)	1649 (43.18)	1376 (39.30)	1135 (31.03)	
**Sex**						< 0.001
Male	6257 (43.89)	1143 (34.86)	1575 (41.24)	1579 (45.10)	1960 (53.58)	
Female	8000 (56.11)	2136 (65.14)	2244 (58.76)	1922 (54.90)	1698 (46.42)	
**Age(years)**						< 0.001
<75	3105 (21.78)	490 (14.94)	748 (19.59)	793 (22.65)	1074 (29.36)	
≥ 75	11,152 (78.22)	2789 (85.06)	3071 (80.41)	2708 (77.35)	2584 (70.64)	
**Residence area**						< 0.001
Urban	3278 (22.99)	231 (7.04)	430 (11.26)	816 (23.31)	1801 (49.23)	
Town	4669 (32.75)	1229 (37.48)	1377 (36.06)	1212 (34.62)	851 (23.26)	
Rural	6310 (44.26)	1819 (55.47)	2012 (52.68)	1473 (42.07)	1006 (27.50)	
**Co-residence type** ^**a**^						< 0.001
Alone	2247 (15.76)	725 (22.40)	654 (17.39)	470 (13.64)	398 (11.01)	
Not alone (in a institution/with household members)	11,813 (82.86)	2512 (77.60)	3106 (82.61)	2977 (86.36)	3218 (88.99)	
**Marital status** ^**b**^						< 0.001
Married/cohabiting	5753 (40.35)	1011 (31.20)	1438 (38.02)	1421 (40.94)	1883 (51.87)	
Divorced/separated/widowed/never married	8370 (58.71)	2229 (68.80)	2344 (61.98)	2050 (59.06)	1747 (48.13)	
**Economic status** ^**c**^						< 0.001
Low	1484 (10.41)	662 (20.44)	416 (11.00)	261 (7.53)	145 (3.99)	
Middle	9891 (69.38)	2256 (69.67)	2771 (73.25)	2461 (71.05)	2403 (66.18)	
High	2741 (19.23)	320 (9.88)	596 (15.75)	742 (21.42)	1083 (29.83)	
**Education level** ^**d**^						< 0.001
Illiterate	6047 (42.41)	1919 (68.98)	1848 (58.30)	1401 (47.25)	879 (26.70)	
Primary school	3856 (27.05)	708 (25.45)	1003 (31.64)	1023 (34.50)	1122 (34.08)	
Middle school	1861 (13.05)	143 (5.14)	288 (9.09)	449 (15.14)	981 (29.80)	
High school or above	445 (3.12)	12 (0.43)	31 (0.98)	92 (3.10)	310 (9.42)	
**Smoking status** ^**e**^						< 0.001
Never	9861 (69.17)	2444 (75.62)	2699 (71.61)	2405 (69.87)	2313 (64.36)	
Former smoker	2071 (14.53)	359 (11.11)	506 (13.43)	484 (14.06)	722 (20.09)	
Current smoker	2105 (14.76)	429 (13.27)	564 (14.96)	553 (16.07)	559 (15.55)	
**Drinking status** ^**f**^						< 0.001
Never	10,363 (72.69)	2543 (78.78)	2816 (74.99)	2510 (73.09)	2494 (69.74)	
Former	1629 (11.43)	353 (10.94)	454 (12.09)	390 (11.36)	432 (12.08)	
Current	2001 (14.04)	332 (10.29)	485 (12.92)	534 (15.55)	650 (18.18)	
**Physical activity** ^**g**^						< 0.001
Never	8638 (60.59)	2463 (76.47)	2630 (69.93)	2082 (60.33)	1463 (40.59)	
Former	1101 (7.72)	180 (5.59)	234 (6.22)	290 (8.40)	397 (11.02)	
Current	4298 (30.15)	578 (17.94)	897 (23.85)	1079 (31.27)	1744 (48.39)	
**Health status** ^**h**^						< 0.001
Surprisingly healthy	3314 (23.24)	461 (14.78)	795 (21.45)	827 (24.25)	1231 (34.51)	
Relative healthy	8405 (58.95)	1975 (63.30)	2292 (61.85)	2126 (62.33)	2012 (56.41)	
Ill	2085 (14.62)	684 (21.92)	619 (16.70)	458 (13.43)	324 (9.08)	
**Hypertension** ^**i**^						
Yes	5479 (41.35)	1106 (37.43)	1299 (36.80)	1365 (41.57)	1709 (49.11)	< 0.001
No	7770 (58.65)	1849 (62.57)	2231 (63.20)	1919 (58.43)	1771 (50.89)	
**Diabetes** ^**j**^						
Yes	1273 (9.96)	145 (5.12)	228 (6.64)	336 (10.60)	564 (16.90)	< 0.001
No	11,504 (90.04)	2687 (94.88)	3208 (93.36)	2835 (89.40)	2774 (83.10)	
**Heart disease** ^**k**^						
Yes	2200 (17.16)	385 (13.50)	445 (12.95)	571 (17.93)	799 (23.85)	< 0.001
No	10,622 (82.84)	2466 (86.50)	2992 (87.05)	2613 (82.07)	2551 (76.15)	
**Stroke and CVD** ^**l**^						
Yes	1482 (11.61)	301 (10.57)	295 (8.59)	404 (12.81)	482 (14.46)	< 0.001
No	11,288 (88.39)	2547 (89.43)	3139 (91.41)	2751 (87.19)	2851 (85.54)	
**Cancer** ^**m**^						
Yes	179 (1.48)	27 (1.01)	26 (0.80)	49 (1.64)	77 (2.46)	< 0.001
No	11,881 (98.52)	2648 (98.99)	3243 (99.20)	2942 (98.36)	3048 (97.54)	
**BMI** ^**n**^						
< 18.5	2167 (16.44)	644 (21.96)	666 (18.80)	502 (15.43)	355 (10.29)	< 0.001
18.5 ~ 23.9	6822 (51.76)	1573 (53.65)	1898 (53.57)	1703 (52.34)	1648 (47.77)	
24.0 ~ 27.9	1031 (7.82)	170 (5.80)	269 (7.59)	243 (7.47)	349 (10.12)	
≥ 28.0	3159 (23.97)	545 (18.59)	710 (20.04)	806 (24.77)	1098 (31.83)	

Data are presented as median(IQR) or n(%)

*IQR* interquartile range, *HEI* healthy eating index, *BMI* body mass index

^a^Data missing for 197 participants

^b^Data missing for 134 participants

^c^Data missing for 141 participants

^d^Data missing for 2048 participants

^e^Data missing for 220 participants

^f^Data missing for 264 participants

^g^Data missing for 220 participants

^h^Data missing for 453 participants

^i^Data missing for 1008 participants

^j^Data missing for 1480 participants

^k^Data missing for 1435 participants

^l^Data missing for 1487 participants

^m^Data missing for 2197 participants

^n^Data missing for 1078 participants

The median (interquartile range) HEI scores for each quartile were as follows: Q1, 17 (15–19); Q2, 23 (22–24); Q3, 28 (27–29); and Q4, 34 (32–37). Participants in higher HEI quartiles were more likely to be male, aged 75 years or older, economically advantaged, physically active, married or cohabiting, urban residents, living with household members or in institutional settings, had higher levels of education, had higher BMI and no chronic diseases such as hypertension, diabetes, heart disease, stroke and cancer.

The percentage distribution of consumption frequency categories for fruit and vegetable across different HEI quartile groups and sarcopenia groups were presented in supplementary Fig. 1 and supplementary Fig. 2. 49.0% and 81.6% of participants in the highest HEI quartile (Q4) reported consuming fruits and vegetables “almost every day,” respectively, which was significantly higher than the proportion observed in the lowest HEI quartile (Q1) group (*P* < 0.001). Additionally, individuals with sarcopenia exhibited lower daily intake frequencies of both vegetables and fruits (*P* < 0.001). See Supplementary Figs. 3–4 for consumption frequency distributions of the remaining 11 foods.

### Association of HEI with sarcopenia

As shown in Table 1, the overall prevalence of sarcopenia was 41.00% and with highest prevalence in Q1 of HEI (*P* < 0.001). Table 2 presents the association between HEI and sarcopenia. Each 1-unit increase in HEI was significantly associated with a 4.4% reduction in prevalence of sarcopenia (OR = 0.956, 95% CI: 0.950–0.962, *P* < 0.001) in Model 1. This relationship remained significant after adjusting for sociodemographic factors in Model 2 (OR = 0.971, 95% CI: 0.963–0.979, *P* < 0.001). After further adjustment for lifestyle and health-related factors in Model 3, each 1-unit increase in HEI was associated with a 1.2% reduction in prevalence of sarcopenia (OR = 0.988, 95% CI: 0.979–0.998, *P* = 0.014).

**Table 2:** The association between HEI and sarcopenia with older participants

	Model 1^a^ OR (95% CI)	Model 2^b^ OR (95% CI)	Model 3^c^ OR (95% CI)	Model 4^d^ OR (95% CI)
**HEI** (per 1 score)	0.956 (0.950–0.962)	0.971 (0.963–0.979)	0.988 (0.979–0.998)	0.978 (0.971–0.985)
*p* value	< 0.001	< 0.001	0.014	< 0.001
**Quartiles of HEI**				
Q1	ref	ref	ref	ref
Q2	0.772 (0.681–0.876)	0.852 (0.736–0.987)	0.934(0.796–1.095)	0.910 (0.807–1.026)
Q3	0.629 (0.552–0.715)	0.737 (0.632–0.860)	0.839 (0.709–0.992)	0.859 (0.756–0.976)
Q4	0.444 (0.390–0.505)	0.581 (0.492–0.687)	0.772 (0.641–0.930)	0.822 (0.711–0.950)
*p for* trend	< 0.001	< 0.001	0.004	0.005

Data are presented as OR (95% CI)

*OR* odds ratio, *CI* confidence interval, *HEI* healthy eating index, *Q1* 1st quartile (≤20), *Q2* 2nd quartile (21-25), *Q3* 3rd quartile (26-30), *Q4* 4th quartile (≥31)

^a^model 1 was unadjusted

^b^model 2 was adjusted for social-demographic co-variables including age, sex, residence area, co-residence type, economic status, marital status and education level

^c^model 3 was adjusted for social-demographic co-variables in model 2 and health-related factors including smoking, drinking, physical activity, health status, BMI and history of diseases

^d^model 4 was using multiple insert data adjusted for co-variables in model 3

In Model 3, analysis by HEI quartiles showed that higher HEI was associated with a lower prevalence of sarcopenia. Compared to Q1, the ORs for sarcopenia were 0.934 (95% CI: 0.796–1.095) for Q2, 0.839 (95% CI: 0.709–0.992) for Q3, and 0.772 (95% CI: 0.641–0.930) for Q4, with a significant decreasing trend (*P*-trend = 0.004). The logistic regression results based on five imputed datasets (Table S2), as well as their pooled results (model 4), were consistent with those of the original dataset, and the analysis of the imputed data served as a sensitivity analysis, indicating the robustness and stability of the results.

**Fig. 2:**
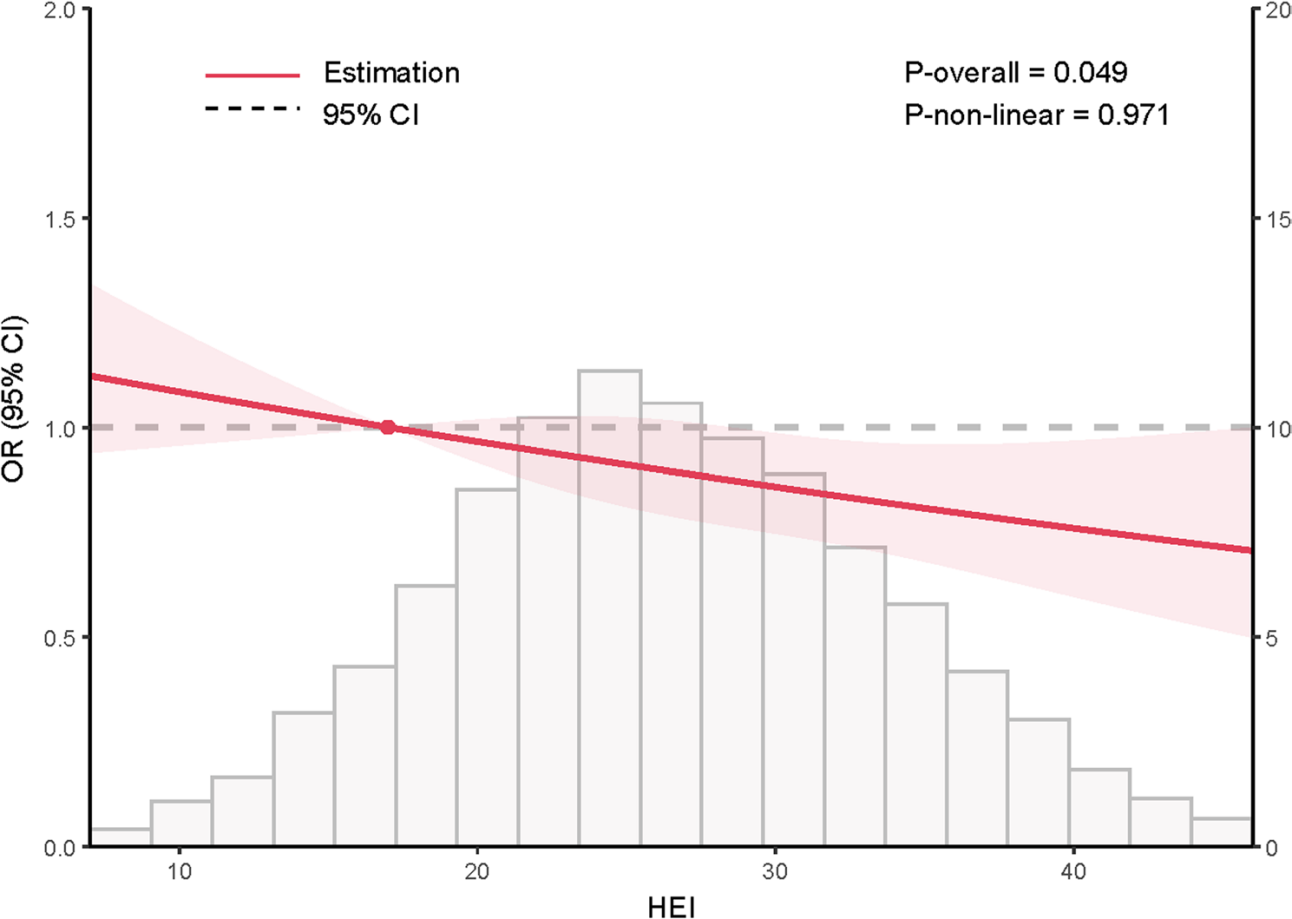
Nonlinear association between the Healthy Eating Index (HEI) and prevalence of sarcopenia among participants in the CLHLS 2018. OR, odds ratio; 95% CI, 95% confidence interval

Restricted cubic splines analysis (Fig. 2) confirmed a linear, inverse association between HEI and prevalence of sarcopenia in overall population (*P*-overall = 0.049, *P*-nonlinear = 0.971).

### Subgroup analysis of the HEI-Sarcopenia association

Subgroup analysis assessed the consistency of the HEI-sarcopenia association across factors such as age, sex, marital status, residential location, co-residence type, economic status and physical activity (Table 3, Table S1). The association varied across subgroups.

In individuals aged 75 years and older, after adjusting for covariates in Model 3, the ORs were 0.939 (95% CI: 0.787–1.121) for Q2, 0.951 (95% CI: 0.794–1.139) for Q3, and 0.793 (95% CI: 0.652–0.964) for Q4, showing a significant decreasing trend across increasing quartiles (*P*-trend = 0.027). A similar association was observed in males, married/cohabiting individuals, those living in institutions or with household members and those never taking physical activities.

**Table 3:** Association between HEI and sarcopenia by prespecified subgroup (original data)^a^

Characteristics	Quartiles of HEI				*P* for trend	*P*-interaction
	Q1	Q2	Q3	Q4		
**Age groups**						0.596
< 75	ref	1.228 (0.696–2.167)	0.560 (0.280–1.121)	1.226 (0.614–2.447)	0.990	
≥ 75	ref	0.939 (0.787–1.121)	0.951 (0.794–1.139)	0.793 (0.652–0.964)	0.027	
**Sex**						0.288
Male	ref	0.773 (0.602–0.993)	0.755 (0.579–0.985)	0.670 (0.499–0.900)	0.009	
Female	ref	0.993 (0.804–1.226)	1.006 (0.808–1.252)	0.916 (0.719–1.167)	0.537	
**Marital status**						0.206
Married/cohabiting	ref	0.927 (0.699–1.231)	0.800 (0.589–1.087)	0.671 (0.474–0.950)	0.018	
Divorced/separated/widowed/never married	ref	0.968 (0.792–1.183)	0.986 (0.797–1.220)	0.883 (0.711–1.096)	0.279	
**Residential area**						0.918
Urban	ref	0.856 (0.601–1.220)	0.884 (0.628–1.244)	0.668 (0.463–0.964)	0.041	
Town	ref	0.864 (0.657–1.137)	0.946 (0.703–1.272)	0.825 (0.608–1.119)	0.300	
Rural	ref	0.980 (0.764–1.259)	0.946 (0.738–1.214)	0.841 (0.653–1.084)	0.166	
**Co-residence type**						0.571
Alone	ref	1.122 (0.770–1.637)	1.152 (0.759–1.748)	1.204 (0.790–1.837)	0.403	
Not alone (in a institution/with household members)	ref	0.868 (0.724–1.041)	0.787 (0.652–0.950)	0.714 (0.581–0.877)	0.001	
**Economic status**						0.614
Low	ref	0.700 (0.396–1.238)	0.818 (0.477–1.403)	0.592 (0.334–1.050)	0.119	
Middle	ref	0.867 (0.713–1.054)	0.915 (0.756–1.108)	0.789 (0.640–0.971)	0.043	
High	ref	1.126 (0.755–1.681)	0.977 (0.664–1.439)	0.899 (0.570–1.419)	0.565	
**Physical activity**						0.751
Never	ref	0.845 (0.685–1.042)	0.834 (0.683–1.020)	0.728 (0.583–0.907)	0.006	
Former	ref	1.260 (0.665–2.389)	1.000 (0.526–1.902)	0.603 (0.307–1.186)	0.074	
Current	ref	1.188 (0.880–1.603)	0.899 (0.644–1.256)	1.066 (0.740–1.534)	0.940	

^a^Adjusted for sociodemographic, lifestyle and health-related covariates (same as in model 3), including age, sex, residence area, co-residence type, economic status, marital status, education level, smoking, drinking, physical activity, health status, BMI and history of diseases

**Fig. 3:**
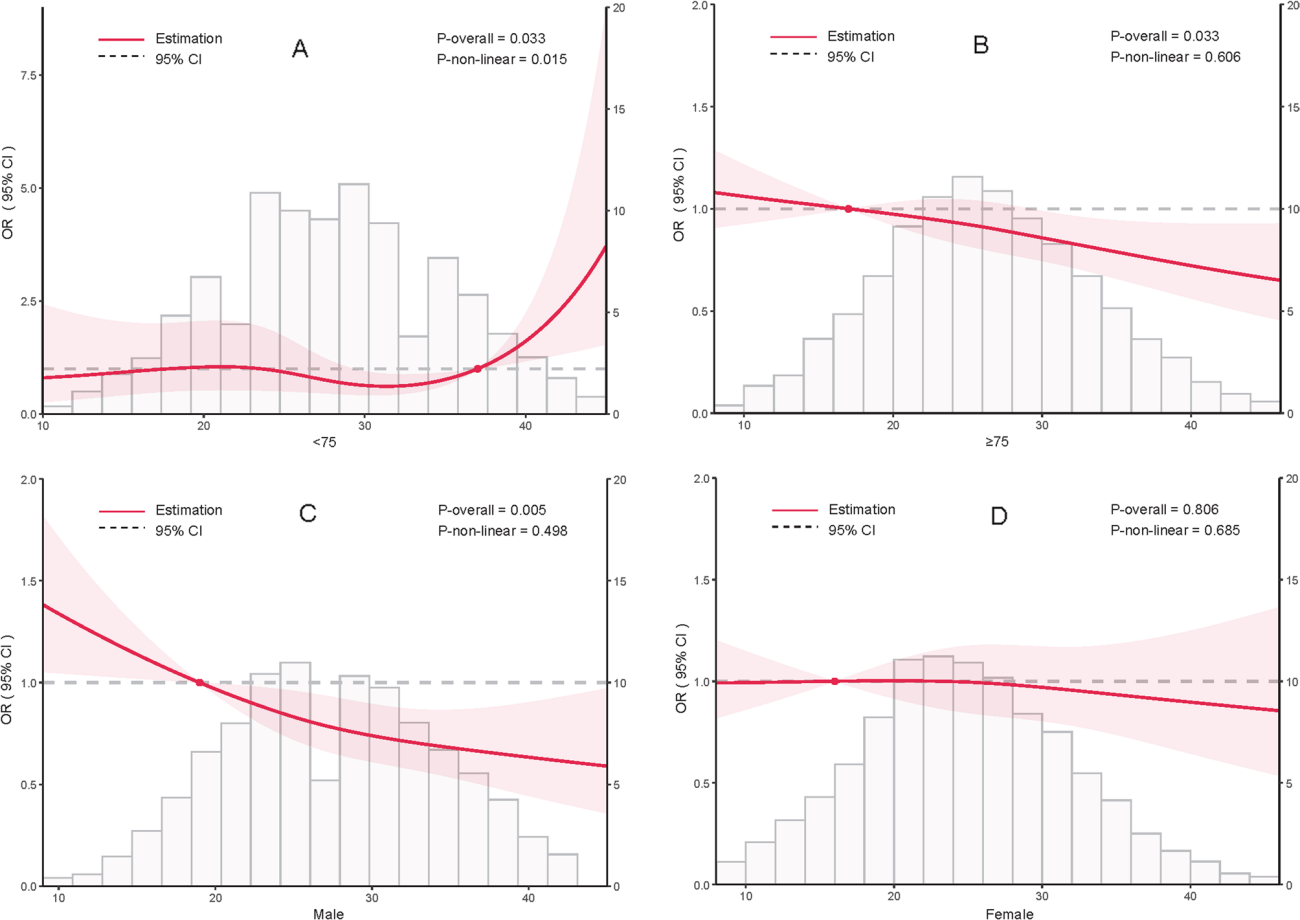
**A-D **Nonlinear relationship between the Healthy Eating Index (HEI) and prevalence of sarcopenia, analyzed using cubic splines, across different subgroups in the CLHLS 2018. OR, odds ratio; 95% CI, 95% confidence interval

Restricted cubic splines analysis (Fig. 3A-D) showed a linear, negative association between HEI and sarcopenia in males, females, and individuals aged 75 or older. Although in subgroup of participants under 75 years older, the association between quartiles of HEI and prevalence of sarcopenia was no significant (*P*-trend = 0.990), but the splines indicated the association might be nonlinear (*P*-overall = 0.033, *P*-nonlinear = 0.015).

## Discussion

In this cross-sectional study of 14,257 participants from a nationally representative sample across 23 provinces in China, we investigated the association between diet quality, measured by the HEI, and sarcopenia in older adults. Our findings demonstrated a significant inverse relationship between HEI scores and prevalence of sarcopenia, even after accounting for sociodemographic, lifestyle, and health-related factors. A dose-response pattern emerged, with higher HEI scores associated with a progressively lower likelihood of sarcopenia.

Sarcopenia, characterized by the progressive loss of skeletal muscle mass and function, leads to diminished muscle strength and physical performance with age. In our study, the overall prevalence of sarcopenia was 41.00%, as determined by the SARC-F questionnaire - the Asian Working Group for Sarcopenia (AWGS) -recommended screening tool for case identification [29]. In a meta-analysis the sensitivity of SARC-F ranged from 27 to 77%, the specificity ranged from 63 to 91%, and the area under the curve (AUC) ranged from 67 to 75%. Nevertheless, it has been proven to be a useful tool for detecting severe cases in community [19, 20]. Given its substantial impact on the elderly, understanding the underlying causes and risk factors is critical. Key contributors to sarcopenia include sedentary behavior, poor nutrition, hormonal changes, and chronic health conditions [30]. These contributors often interact synergistically, compounding the risk and accelerating muscle loss. Therefore, identifying modifiable risk factors, such as diet quality, is crucial for developing effective prevention and management strategies for sarcopenia in older adults.

The HEI, used as a proxy for diet quality in our study, was calculated based on the consumption frequency of 13 food groups. It is a widely recognized index that reflects the overall quality and diversity of an individual’s diet, with positive scores assigned to health-promoting foods and negative scores to health-damaging foods [16, 17]. The inverse association observed between HEI scores and sarcopenia risk in our study is consistent with findings from various populations [31]. A study using National Health and Nutrition Examination Survey(NHANES)data found that participants in the highest quartile of the HEI-2015 had a significantly lower risk of sarcopenia compared to those in the lowest quartile (OR = 0.76, 95% CI: 0.60–0.96) [31]. Similarly, a cross-sectional study in Iran demonstrated that older adults in the highest HEI-2015 quartile had 69% lower odds of probable sarcopenia (OR = 0.31, 95% CI: 0.12–0.81, *P*-trend = 0.03) compared to those in the lowest quartile. In this study, a significant positive association between HEI scores and handgrip strength was also found, further supporting the relationship between diet quality and muscle health [13]. Subgroup analyses revealed differential associations between HEI and sarcopenia prevalence. The protective effect was particularly pronounced in adults aged ≥ 75 years, potentially reflecting increased nutritional vulnerability in advanced age. Similarly, stronger associations were observed among those not living alone suggesting social support may enhance dietary benefits [32, 33]. These findings on the association between HEI and sarcopenia underscore the pivotal role of dietary pattern modification in mitigating age-related muscle loss. For older individuals with suboptimal HEI scores, targeted dietary interventions focusing on improving overall diet quality may represent a feasible, cost-effective primary prevention strategy against sarcopenia development.

However, a study among Iranian elders conducted by different researchers reported mixed findings, potentially due to sample size limitations. While no significant association was found between the AHEI-2010 and sarcopenia risk in men or the general population, a significant protective association was identified among women (OR = 0.20, 95% CI: 0.04–0.91, *P*-trend = 0.039) [14]. This discrepancy may reflect variations in population characteristics or regional dietary patterns, highlighting the need for further research to confirm these associations across diverse populations.

Although studies specifically examining the association between the Healthy Eating Index (HEI) and sarcopenia are limited, there is a substantial body of research on the relationship between diet quality and sarcopenia. A systematic review analyzed 14 prospective cohort studies and found that adherence to the Mediterranean diet significantly reduced the risk of sarcopenia in European populations, although this association was not observed in non-European populations [34]. Other dietary pattern scores, such as the 12-component revised Japanese Diet Index (rJDI12), the Japanese Food Guide Spinning Top (JFG-ST), the dietary variety score (DVS), and the dietary quality index-international (DQI-I), have also been explored in relation to sarcopenia, though further studies are needed to validate their associations [35, 36].

In both Chinese and American food guidelines, the intake of certain food groups, such as fresh fruits and vegetables, is considered beneficial for muscle health. However, the evidence regarding these foods’ association with sarcopenia is inconsistent and warrants further investigation. A study in a Hong Kong Chinese community-dwelling population found that men with higher “vegetables-fruits” dietary pattern scores had a lower likelihood of sarcopenia compared with those in the lowest quartile of the “vegetables-fruits” pattern [35]. Similarly, a cross-sectional study using data from the Fourth Korea National Health and Nutrition Examination Survey (KNHANES) found that the consumption of vegetables, fruits, or both was inversely associated with sarcopenia in men, but for women, only those with high fruit consumption had a lower risk of sarcopenia [37]. Furthermore, a study across six low- and middle-income countries revealed that fruit consumption was associated with a reduced risk of sarcopenia in females and the overall population, but no significant associations were found among older males [8]. In our study, participants in the highest quartile (Q4) of HEI demonstrated significantly higher consumption frequencies of both fruits and vegetables, whereas individuals with sarcopenia showed markedly lower intake frequencies for these food groups. These findings suggest that specific dietary patterns and food groups, particularly fruits and vegetables, may play myoprotective effect through modulating redox balance, affecting signaling pathways and transcriptional factors or reducing age-related chronic disease [38]. But the evidence remains inconsistent across different populations and many further researches were needed to clarify the nature and strength of these associations and to determine potential cultural or demographic factors that may influence their effectiveness.

### Strengths and limitations

This study utilized the 2018 CLHLS cross-sectional data, a nationally representative, large-scale survey of the elderly Chinese population, encompassing over 10,000 individuals. The substantial sample size enhances the credibility and generalizability of our findings. However, several limitations should be noted. First, as a cross-sectional study, it is not possible to establish a causal relationship between HEI and the risk of sarcopenia. Second, The HEI was derived from self-reported food frequency data, which inherently lacks quantitative assessment of actual portion sizes for specific food groups. This methodological limitation may lead to an incomplete representation of true dietary quality, thereby introducing potential bias in subsequent analyses. Third, while our adapted HEI maintained the core principles of diet quality assessment, certain modifications were necessary due to data constraints in the CLHLS dataset. This adaptation, though consistent with prior studies using similar Chinese population data [16, 17], may affect direct comparability with standard HEI versions. Fourth, while we adjusted for sociodemographic factors, lifestyle, and health status including BMI, some important covariates such as waist circumference (a potential marker of central adiposity) and detailed medication use were not available in the CLHLS dataset. Additionally, some included covariates may not have been measured with optimal precision. These unmeasured and imperfectly measured factors could potentially influence the observed associations. Finally, while the SARC-F questionnaire employed in this study demonstrates utility as a pragmatic screening instrument for sarcopenia, its diagnostic sensitivity remains limited for definitive confirmation of the condition.

## Conclusion

This study establishes a significant inverse association between HEI and the prevalence of sarcopenia in the older Chinese population, with a consistent linear relationship observed across both genders. These findings highlight the pivotal role of dietary quality in maintaining muscle mass and function in older adults. In light of the growing global prevalence of sarcopenia, our results emphasize the urgent need for the promotion of balanced, nutrient-dense diets as a primary preventive strategy. Healthcare providers, especially in geriatric care settings, along with family caregivers, should prioritize dietary interventions that focus on food diversity and quality to reduce the risk of sarcopenia. This study offers important implications for future public health efforts aimed at enhancing nutrition among older adults, with the potential to substantially reduce the burden of sarcopenia and its associated health consequences.

## Supplementary Information

Supplementary Material 1.

Supplementary Material 2.

Supplementary Material 3.

Supplementary Material 4.

## Data Availability

The dataset that supports the conclusions of this article can be found in the domain of the CLHLS and is accessible at http://opendata.pku.edu.cn/.

## Abbreviations

HEI: Healthy Eating Index
CLHLS: Chinese Longitudinal Healthy Longevity Survey
ORs: Odds Ratio
BMI: Body Mass Index
95% CIs: 95% Confidence Intervals
IQR: Interquartile Range
AHEI-2010: Alternative Healthy Eating Index-2010

## References

[1] Cruz-Jentoft AJ, Bahat G, Bauer J, Boirie Y, Bruyere O, Cederholm T, Cooper C, Landi F, Rolland Y, Sayer AA, et al. Sarcopenia: revised European consensus on definition and diagnosis. Age Ageing. 2019;48(1):16–31. 30312372 10.1093/ageing/afy169 PMC6322506

[2] Yuan S, Larsson SC. Epidemiology of sarcopenia: prevalence, risk factors, and consequences. Metabolism. 2023;144:155533. 36907247 10.1016/j.metabol.2023.155533

[3] Xia L, Zhao R, Wan Q, Wu Y, Zhou Y, Wang Y, Cui Y, Shen X, Wu X. Sarcopenia and adverse health-related outcomes: an umbrella review of meta-analyses of observational studies. Cancer Med. 2020;9(21):7964–78. 32924316 10.1002/cam4.3428 PMC7643685

[4] United Nations DoEaSA, Population Division. (2024). World Population Prospects 2024, Online Edition.https://population.un.org/wpp/Download/Standard/Population/. In.

[5] Petermann-Rocha F, Balntzi V, Gray SR, Lara J, Ho FK, Pell JP, Celis-Morales C. Global prevalence of sarcopenia and severe sarcopenia: a systematic review and meta-analysis. J Cachexia Sarcopenia Muscle. 2022;13(1):86–99. 34816624 10.1002/jcsm.12783 PMC8818604

[6] Gajda R, Jezewska-Zychowicz M, Raczkowska E, Rak K, Szymala-Pedzik M, Noculak L, Sobieszczanska M. Association of Dietary Patterns, Suspected Sarcopenia, and Frailty Syndrome among Older Adults in Poland-A Cross-Sectional Study. Nutrients. 2024;16(18). 10.3390/nu16183090 PMC11434646 39339690

[7] Ganapathy A, Nieves JW. Nutrition and Sarcopenia-What do we know? Nutrients. 2020;12(6):1755. 10.3390/nu12061755 PMC7353446 32545408

[8] Koyanagi A, Veronese N, Solmi M, Oh H, Shin JI, Jacob L, Yang L, Haro JM, Smith L. Fruit and vegetable consumption and sarcopenia among older adults in Low- and Middle-Income countries. Nutrients. 2020;12(3):706. 10.3390/nu12030706 PMC7146581 32155879

[9] Hong SH, Bae YJ. Association of dietary vegetable and fruit consumption with sarcopenia: A systematic review and Meta-Analysis. Nutrients. 2024;16(11):1707. 10.3390/nu16111707 PMC11174889 38892640

[10] Kohl J, Hohberg V, Hauff P, Lang C, Faude O, Gollhofer A, Konig D. Development of a metric healthy eating Index-2015 and comparison with the healthy eating Index-2015 for the evaluation of dietary quality. Front Nutr. 2022;9:952223. 36082033 10.3389/fnut.2022.952223 PMC9448016

[11] Kennedy ET, Ohls J, Carlson S, Fleming K. The healthy eating index: design and applications. J Am Diet Assoc. 1995;95(10):1103–8. 7560680 10.1016/S0002-8223(95)00300-2

[12] Papaioannou KG, Nilsson A, Nilsson LM, Kadi F. Healthy eating is associated with sarcopenia risk in physically active older adults. Nutrients. 2021;13(8):2813. 10.3390/nu13082813 PMC8401667 34444973

[13] Esmaeily Z, Tajary Z, Daei S, Rezaei M, Eyvazkhani A, Dorosty Motlagh AR, Palmowski A. Association between healthy eating Index-2015 scores and probable sarcopenia in community-dwelling Iranian older adults: a cross-sectional study. J Nutr Sci. 2021;10:e20. 33996033 10.1017/jns.2021.12 PMC8080184

[14] Ghoreishy SM, Koujan SE, Hashemi R, Heshmat R, Motlagh AD, Esmaillzadeh A. Relationship between healthy eating index and sarcopenia in elderly people. BMC Geriatr. 2023;23(1):25. 36639737 10.1186/s12877-023-03734-3 PMC9840332

[15] Zeng Y, Shi X, Zheng Z, Lei X. [China], 1998–2018. Chinese longitudinal healthy longevity survey (CLHLS). In.: Inter-university Consortium for Political and Social Research [distributor]; 2024.

[16] Zhang X, Zhou W, Wang H, Bai Y, Zhang F, Lu W. Association between healthy eating and depression symptoms among Chinese older adults: A cross-sectional study based on the Chinese longitudinal healthy longevity survey. Prev Med Rep. 2024;38:102616. 38298821 10.1016/j.pmedr.2024.102616 PMC10828603

[17] Yan LL, Li C, Zou S, Li Y, Gong E, He Z, Shao S, Jin X, Hua Y, Gallis JA, et al. Healthy eating and all-cause mortality among Chinese aged 80 years or older. Int J Behav Nutr Phys Act. 2022;19(1):60. 35619133 10.1186/s12966-022-01280-6 PMC9137098

[18] Tan JY, Zeng QL, Ni M, Zhang YX, Qiu T. Association among calf circumference, physical performance, and depression in the elderly Chinese population: a cross-sectional study. BMC Psychiatry. 2022;22(1):278. 35443643 10.1186/s12888-022-03925-z PMC9020001

[19] Yang M, Hu X, Xie L, Zhang L, Zhou J, Lin J, Wang Y, Li Y, Han Z, Zhang D, et al. Comparing Mini sarcopenia risk assessment with SARC-F for screening sarcopenia in Community-Dwelling older adults. J Am Med Dir Assoc. 2019;20(1):53–7. 29909052 10.1016/j.jamda.2018.04.012

[20] Guo JY, Yu K, Li CW, Bao YY, Zhang Y, Wang F, Li RR, Xie HY. The application of Chinese version of SARC-F and SARC-CalF in sarcopenia screening against five definitions: a diagnostic test accuracy study. BMC Geriatr. 2024;24(1):883. 39462351 10.1186/s12877-024-05460-w PMC11515234

[21] Malmstrom TK, Miller DK, Simonsick EM, Ferrucci L, Morley JE. SARC-F: a symptom score to predict persons with sarcopenia at risk for poor functional outcomes. J Cachexia Sarcopenia Muscle. 2016;7(1):28–36. 27066316 10.1002/jcsm.12048 PMC4799853

[22] Chen LK, Woo J, Assantachai P, Auyeung TW, Chou MY, Iijima K, Jang HC, Kang L, Kim M, Kim S, et al. Asian working group for sarcopenia: 2019 consensus update on sarcopenia diagnosis and treatment. J Am Med Dir Assoc. 2020;21(3):300–e307302. 32033882 10.1016/j.jamda.2019.12.012

[23] Wu AH, Setiawan VW, Lim U, Tseng CC, White KK, Shepherd J, Lenz HJ, Cheng I, Stram DO, Haiman C, et al. Prognostic utility of self-reported sarcopenia (SARC-F) in the multiethnic cohort. J Cachexia Sarcopenia Muscle. 2022;13(2):987–1002. 35098697 10.1002/jcsm.12916 PMC8977971

[24] Fan H, Kouvari M, Guo C, Liu Z, Zhang X, Wang H, Li Y, Zhang T, Mantzoros CS. A comprehensive comparison of two commonly used BMI thresholds for non-communicable diseases and Multimorbidity in the Chinese population. Clin Nutr. 2025;48:70–9. 40154198 10.1016/j.clnu.2025.03.016

[25] LaValley MP. Logistic regression. Circulation. 2008;117(18):2395–9. 18458181 10.1161/CIRCULATIONAHA.106.682658

[26] Li X, Schottker B, Holleczek B, Brenner H. Associations of DNA methylation algorithms of aging and cancer risk: results from a prospective cohort study. EBioMedicine. 2022;81:104083. 35636319 10.1016/j.ebiom.2022.104083 PMC9157462

[27] Li X, Schottker B, Holleczek B, Brenner H. Association of longitudinal repeated measurements of frailty index with mortality: cohort study among community-dwelling older adults. EClinicalMedicine. 2022;53:101630. 36119560 10.1016/j.eclinm.2022.101630 PMC9475257

[28] Huang S, Zhong D, Lv Z, Cheng J, Zou X, Wang T, Wen Y, Wang C, Yu S, Huang H, et al. Associations of multiple plasma metals with the risk of metabolic syndrome: A cross-sectional study in the mid-aged and older population of China. Ecotoxicol Environ Saf. 2022;231:113183. 35032729 10.1016/j.ecoenv.2022.113183

[29] Voulgaridou G, Tyrovolas S, Detopoulou P, Tsoumana D, Drakaki M, Apostolou T, Chatziprodromidou IP, Papandreou D, Giaginis C, Papadopoulou SK. Diagnostic criteria and measurement techniques of sarcopenia: A critical evaluation of the Up-to-Date evidence. Nutrients. 2024;16(3):436. 10.3390/nu16030436 PMC10856900 38337720

[30] Cho MR, Lee S, Song SK. A review of sarcopenia pathophysiology, diagnosis, treatment and future direction. J Korean Med Sci. 2022;37(18):e146. 35535373 10.3346/jkms.2022.37.e146 PMC9091430

[31] Bigman G, Ryan AS. Healthy eating Index-2015 is associated with grip strength among the US adult population. Nutrients. 2021;13(10):3358. 10.3390/nu13103358 PMC8540420 34684359

[32] Long T, Zhang K, Chen Y, Wu C. Trends in diet quality among older US adults from 2001 to 2018. JAMA Netw Open. 2022;5(3):e221880. 35275167 10.1001/jamanetworkopen.2022.1880 PMC8917422

[33] Pieroth R, Rigassio Radler D, Guenther PM, Brewster PJ, Marcus A. The relationship between social support and diet quality in Middle-Aged and older adults in the united States. J Acad Nutr Diet. 2017;117(8):1272–8. 28483451 10.1016/j.jand.2017.03.018

[34] Jang EH, Han YJ, Jang SE, Lee S. Association between diet quality and sarcopenia in older adults: systematic review of prospective cohort studies. Life (Basel). 2021;11(8):811. 10.3390/life11080811 PMC8399213 34440555

[35] Chan R, Leung J, Woo J. A prospective cohort study to examine the association between dietary patterns and sarcopenia in Chinese Community-Dwelling older people in Hong Kong. J Am Med Dir Assoc. 2016;17(4):336–42. 26774365 10.1016/j.jamda.2015.12.004

[36] Shimizu A, Okada K, Tomata Y, Uno C, Kawase F, Momosaki R. Association between Japanese diet adherence and muscle weakness in Japanese adults aged >/=50 years: findings from the JSTAR cohort study. Int J Environ Res Public Health. 2023;20(22):7065. 10.3390/ijerph20227065 PMC10671671 37998296

[37] Kim J, Lee Y, Kye S, Chung YS, Kim KM. Association of vegetables and fruits consumption with sarcopenia in older adults: the fourth Korea National health and nutrition examination survey. Age Ageing. 2015;44(1):96–102. 24646604 10.1093/ageing/afu028

[38] Granic A, Sayer AA, Robinson SM. Dietary patterns, skeletal muscle health, and sarcopenia in older adults. Nutrients. 2019;11(4):745. 10.3390/nu11040745 PMC6521630 30935012

