# Supplementary figures and images for: Association between the healthy eating index and sarcopenia in Chinese elderly: a cross-sectional study
Source: BMC Geriatr. 2025 Jul 5;25:494. doi: 10.1186/s12877-025-06143-w (PMC12228325; doi:10.1186/s12877-025-06143-w)

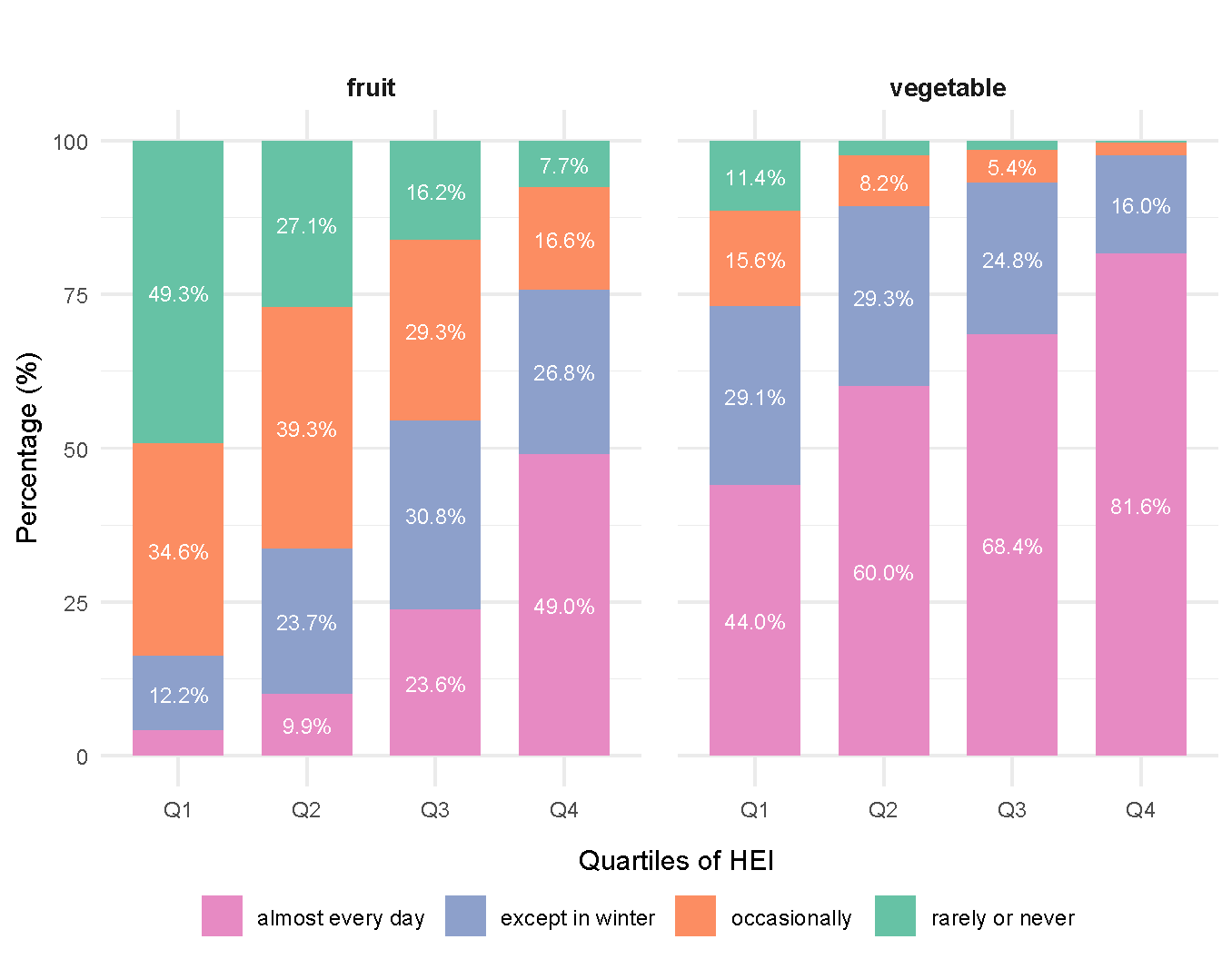

Supplement: Supplementary file 1 — Supplementary Material 1. [file 12877_2025_6143_MOESM1_ESM.tiff]

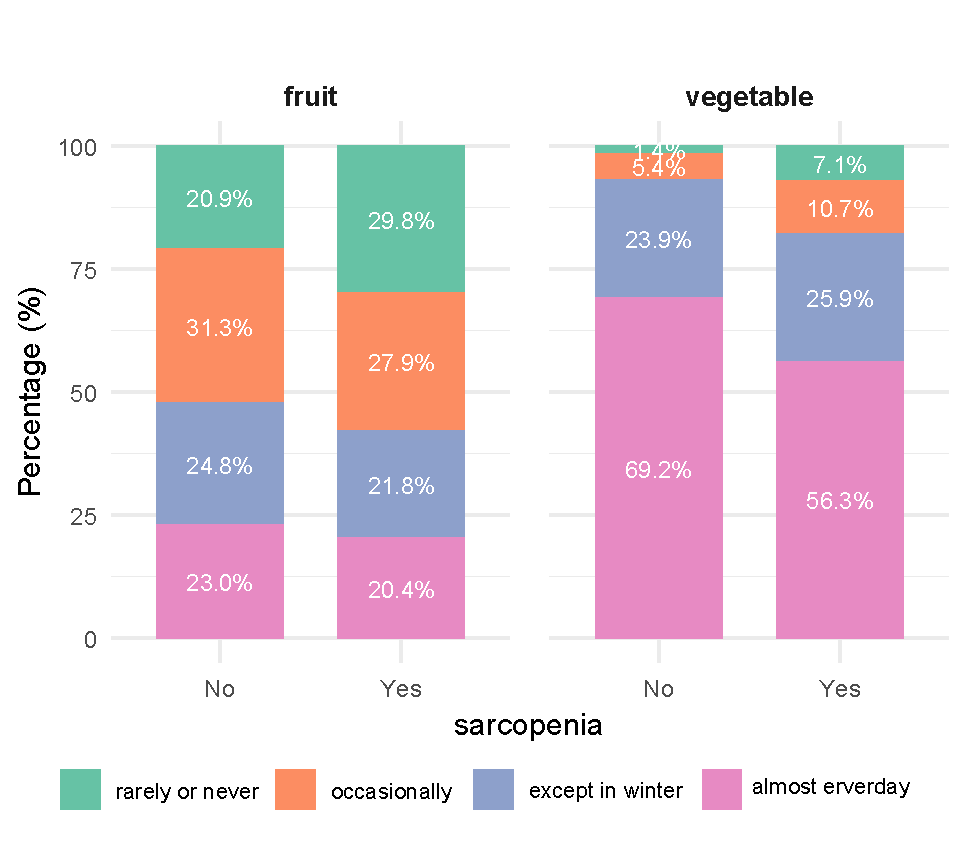

Supplement: Supplementary file 2 — Supplementary Material 2. [file 12877_2025_6143_MOESM2_ESM.tiff]

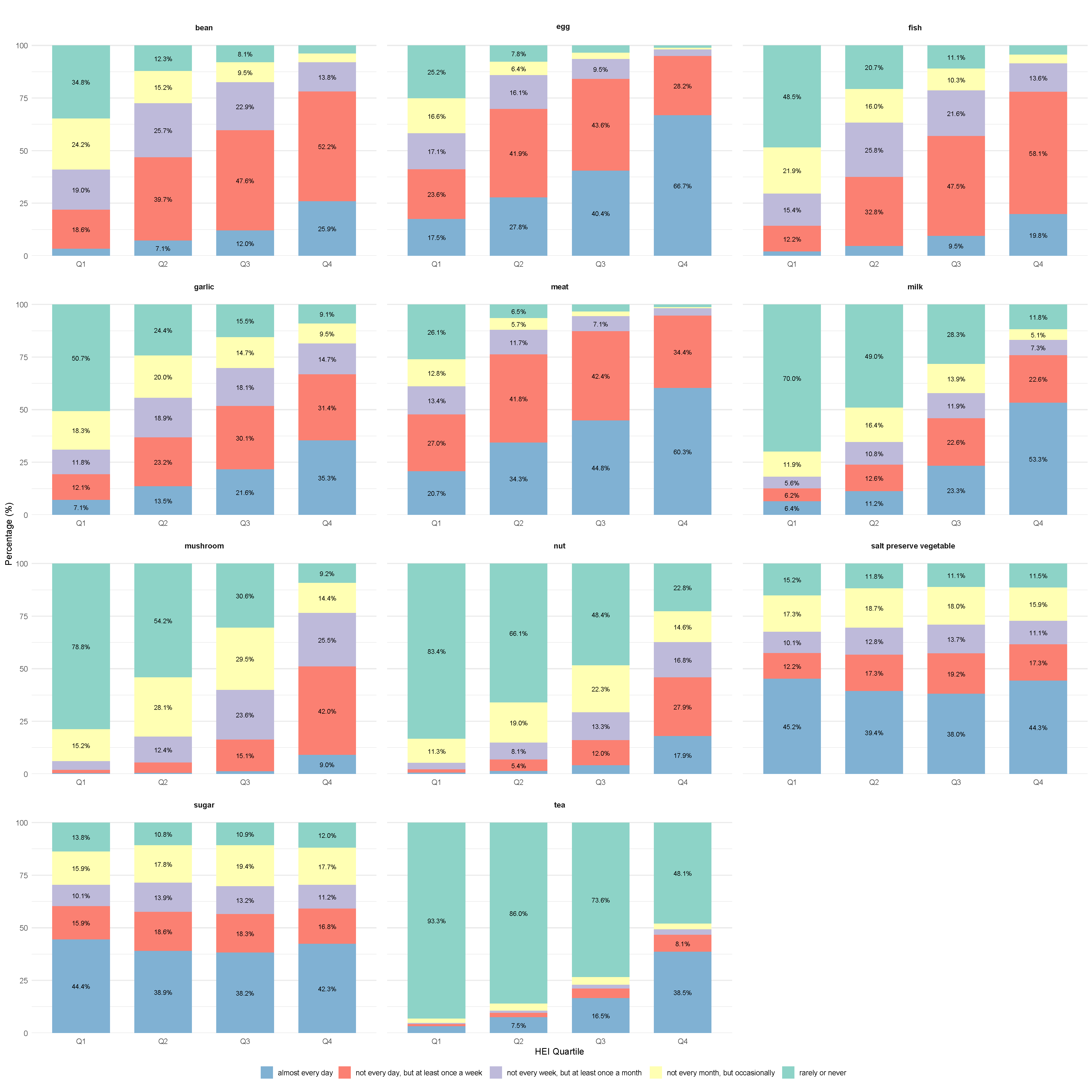

Supplement: Supplementary file 3 — Supplementary Material 3. [file 12877_2025_6143_MOESM3_ESM.tiff]

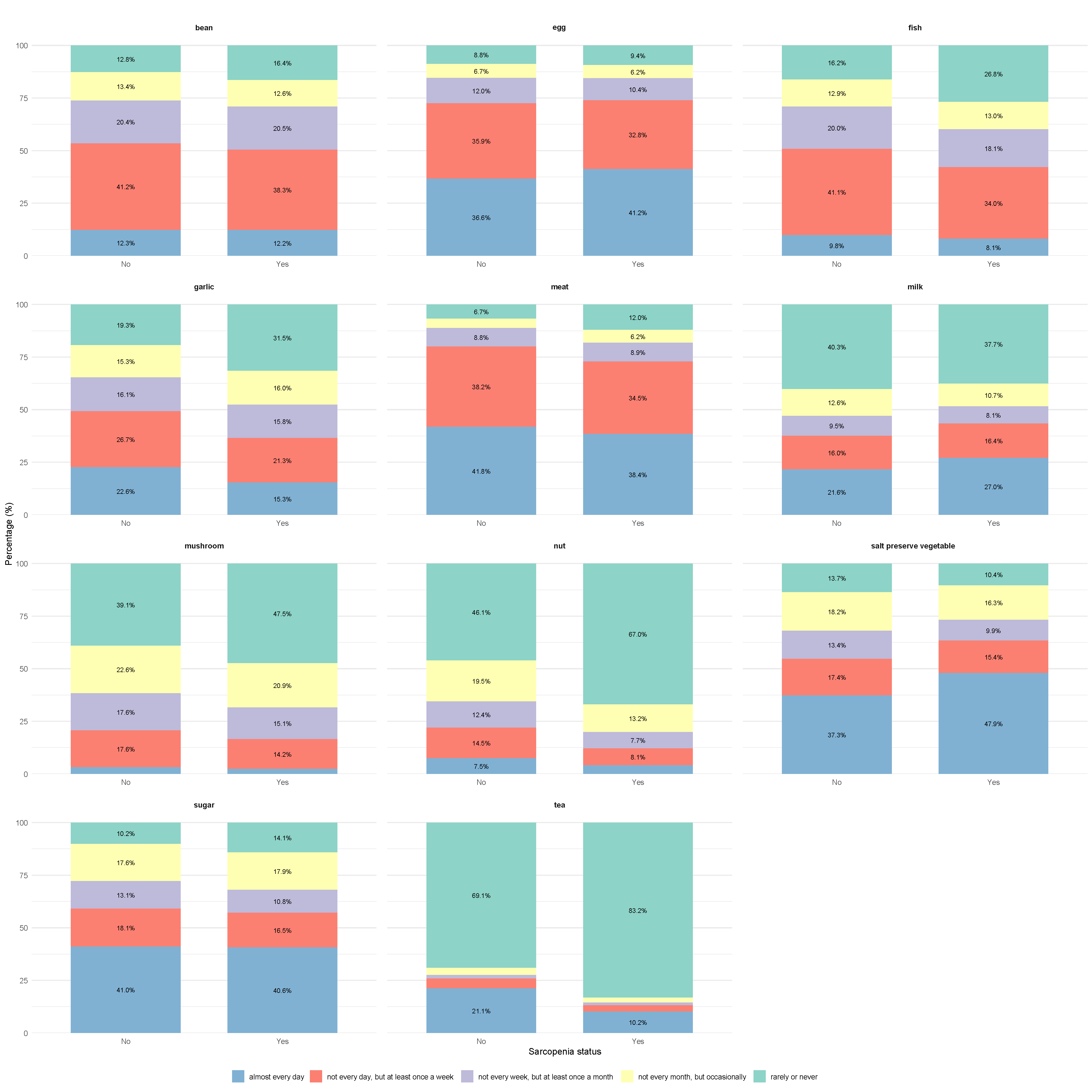

Supplement: Supplementary file 4 — Supplementary Material 4. [file 12877_2025_6143_MOESM4_ESM.tif]
